# CRISPR/Cas9 mediated ENT2 gene knockout altered purine catabolic pathway and induced apoptosis in colorectal cell lines

**DOI:** 10.1371/journal.pone.0329501

**Published:** 2025-08-18

**Authors:** Safaa M. Naes, Sharaniza Ab-Rahim, Musalmah Mazlan, Saiful Effendi Syafruddin, M. Aiman Mohtar, Asmaa Y. Abuhamad, Amirah Abdul Rahman

**Affiliations:** 1 Department of Biochemistry and Molecular Medicine, Faculty of Medicine, Universiti Teknologi MARA, Cawangan Selangor, Kampus Sungai Buloh, Sungai Buloh, Selangor, Malaysia; 2 Faculty of Medicine, Institute of Medical and Molecular Biotechnology, Universiti Teknologi MARA, Cawangan Selangor, Kampus Sungai Buloh, Sungai Buloh, Selangor, Malaysia; 3 Faculty of Health and Life Sciences, Management and Science University, Shah Alam, Selangor, Malaysia; 4 UKM Medical Molecular Biology Institute, Universiti Kebangsaan Malaysia, Kuala Lumpur, Malaysia; 5 Bionanotechnology Research Group and Department of Biochemistry, Faculty of Biotechnology and Biomolecular Sciences, Universiti Putra Malaysia, Seri Kembangan, Selangor, Malaysia; Texas A&M University, UNITED STATES OF AMERICA

## Abstract

Although purine metabolism is one of the most impacted pathways in colorectal cancer (CRC), little is known about the role of *equilibrative nucleoside transporter 2* (*ENT2*) in CRC development and its association with the altered purine metabolism pathway. This study aimed to determine the role of *ENT2* in altered purine metabolism in the early and late stages of CRC using CRISPR/Cas9 gene editing tools and a variety of functional experiments. The expression of *ENT2* was significantly higher (P < 0.001) in all CRC cell lines as compared to the normal colon cells. The two CRC cell lines with the highest *ENT2* expression, the early stage HT29 cells and the late stage DLD1 cells, were knocked out (KO) using the CRISPR/Cas9 tool. The hypoxanthine (HPX) level and the xanthine oxidase (XO) activity were significantly higher in both HT29/KO and DLD1/KO single cell-derived clones (P < 0.01). The increase in HPX level and XO activity were associated with an elevation in the reactive oxygen species (ROS) level. These data suggest that the ENT2 KO elevated the ROS levels induced apoptosis and impaired the cell proliferation of the early stage of CRC cell line, i.e., HT29/KO clonal cells. In this context, targeting *ENT2* gene might be a potential strategy in CRC treatment by increasing the production of ROS and hence, inducing the apoptosis pathway.

## Introduction

Based on GLOBOCAN 2022 statistics for cancer incidences, colorectal cancer (CRC) is the third most common cancer in men and the second in women worldwide [1]. In 2020, CRC accounted for an estimated 1.9 million incidence cases and 0.9 million deaths globally [2]. By 2030, more than 2.2 million new cases of CRC and 1.1 million deaths are expected [3]. The exact causes of CRC are still unclear. The revised report of the World Cancer Research Fund/American Institute for Cancer Research revealed cogent evidence implicating that alcoholic drinks, body fatness, and processed meat increase the risk of CRC [4].

Altered metabolism pathway, particularly purine metabolism, has been linked to CRC progression [5,6]. Purines are fundamental building blocks of DNA and RNA and other intracellular modulators. They are vital for producing metabolic energy, synthesizing nucleic acids, and supporting cells proliferation [7,8]. Hypoxanthine (HPX) is a purine nucleobase. It plays a role in nucleic acid synthesis by the salvage pathway. HPX functions as metabolic precursors in the synthesis of nucleic acids and as primary substrates for energy metabolism (ATP and GTP) [9]. It is also the first metabolite derivative in the purine catabolic pathway [10].

In the purine catabolic pathway, HPX is converted to xanthine and uric acid via xanthine oxidase (XO) and generates the reactive oxygen species (ROS) as a by-product [11]. The cytotoxicity of ROS can potentially cause different diseases but may also destroy cancer cells [12], hence making it a potential target for treating the different types of cancers, including CRC. HPX is a hydrophilic molecule, and it requires nucleoside transporters (NTs), particularly the equilibrative nucleoside transporter 2 (ENT2), for its translocation across the cell membranes [13]. ENT2 mediates a wide range of purine and pyrimidine nucleosides/ nucleobases and plays a crucial role in transporting the anticancer nucleoside analogue drugs across the cells [14]. *ENT2* showed the highest mRNA-level expression in the cancers derived from digestive organ tissues [15]. In particular, *ENT2* was highly expressed in colorectal biopsy samples [16], and several metastatic colon cancer cell lines (Colo205, LoVo, SK-CO-1, and T84) and primary cell lines (Caco2, Colo320, HCT116, and HT29) [17]. In another study, *ENT2* overexpressed in four CRC cell lines presenting different stages of CRC implies its importance in facilitating hypoxanthine transport that is required for enhanced DNA synthesis via hypoxanthine recycling [18].

However, the link between the *ENT2* gene expression and purine catabolism remains unexplored. Therefore, this study aimed to target *ENT2*, i.e., the specific transporter for HPX, using the CRISPR/Cas9 gene-editing tool to determine the role of *ENT2* in altered purine metabolism in the early and late stages of CRC cell lines. Also, this study evaluated whether targeting the *ENT2* could serve as a potential strategy for CRC treatment by increasing ROS production, and hence, inducing the apoptosis pathway.

## Materials and methods

### 1. Cell culture

Ethical exemption was received from UiTM Research Ethics Committee (REC) (Ref. No: REC/03/2025 (ST/EX/6)). This study investigated a panel of human CRC cell lines purchased from the American Type Culture Collection (ATCC, Rockville, MD, USA). These cell lines were SW1116, HT29, DLD1, and HCT116 derived from early and late CRC tissues, i.e., Dukes’ A, Dukes’ B, Dukes’ C, and Dukes’ D, respectively, and also the normal colon epithelial cell line, CCD-841CoN. Tumour stages of CRC were classified following Dukes’ classification criteria. For lentivirus production, HEK293T cells (obtained from ATCC, USA) were used. All cell lines were cultured in Dulbecco’s Modified Eagle Medium (DMEM, high glucose, Nacalai Tesque, Japan) supplemented with 10% fetal bovine serum (FBS, Gibco, USA) and 1% penicillin-streptomycin (Sigma-Aldrich, USA). Cells were incubated at 37 °C in a humidified incubator (BINDER, Germany), with 5% CO_2_.

### 2. RNA extraction and cDNA

Total RNA was extracted from the cells using a NucleoSpin™ RNA isolation column kit (Macherey-Nagel, Germany) according to the manufacturer’s recommendations. The concentration and the purity of the extracted RNA were determined using a UV-Vis spectrophotometer (SpectraMax® QuickDrop™, Molecular Devices, USA). Altogether, 250 ng of RNA was reverse-transcribed into cDNA using SensiFAST™ cDNA synthesis kit (Bioline, UK) and following the manufacturer’s protocol.

### 3. Quantitative real-time PCR (qRT-PCR)

The qRT-PCR was performed using the 2x fluorescent qRT-PCR mix (SensiFAST™ SYBR & Fluorescein Mix, Bioline, UK) on a thermal cycler (CFX96™ Real-Time PCR instrument, BioRad, USA). The reaction, containing the 2x fluorescent qRT-PCR mix, 1 µL cDNA, 400 nM of each forward and reverse primer, and DNase/RNase -free water, was prepared in a final volume of 10 µL. All primers used for the qRT-PCR reaction were designed using the algorithm BLAST on the National Center for Biotechnology Information (NCBI) website and purchased from Integrated DNA Technologies (IDT). Primer sequences are listed in S1 Table. The Ct values of the genes of interest were normalised based on the Ct value of the reference genes, *GAPDH* and *HPRT1*. The relative expression ratio of the gene was determined using the 2^-∆∆Ct^ method [19].

### 4. Generation of the CRC cell lines with *ENT2* knockout using the CRISPR/Cas9 gene-editing tool

In this study, five independent single guide RNA (sgRNAs) constructs targeting the *ENT2* gene (hereinafter referred to as sgENT2) and non-targeting control (sgNTC) were designed using the Broad Institute sgRNA designer webtool (https://portals.broadinstitute.org/). These sgENTs constructs were designed to harbour the BbsI restriction site at the 5’ and 3’ ends S2 Table (A and B). The top and bottom strands of sgENTs were purchased separately from IDT. This study used the sgRNA expression vector pKLV-U6gRNA(BbsI)-PGKhygro2AeGFP modified from pKLV-U6gRNA(BbsI)-PGKpuro2ABFP (Addgene Plasmid) [20]. The top and bottom strands were annealed and phosphorylated using the T4 Polynucleotide kinase (New England Biolabs, UK). The annealed sgENT2 constructs were ligated into the Antarctic phosphatase-treated/BbsI-digested plasmid using the T4 ligase (New England Biolabs, UK) at 16 °C overnight, followed by transformation into the chemically competent DH5α Escherichia coli strain (New England Biolabs, UK). The transformed bacteria were then plated on Luria Broth (LB; Sigma-Aldrich, USA), supplemented with 100 μg/mL ampicillin (Sigma-Aldrich, USA), and incubated at 37 °C overnight. On the following day, a single colony of the transformed bacteria was picked up and cultured in 5 mL of LB broth containing ampicillin (100 μg/mL) and incubated overnight at 37 °C with agitation. The presence of ligated sgENT2 in the expression vectors was verified via Sanger sequencing using a U6 promoter forward primer (S3 Fig).

HEK293T cells were transfected with a mixture of 1.5 μg plasmid of interest, psPAX2, and 0.5 μg pMD2.G in the DMEM serum-free media. After 72-hour post-transfection, the media containing lentivirus particles were collected and filtered through a sterile filter of 0.45 μM (Biopure™, USA). CRC Cells (HT29 and DLD1) were transduced with the lentiviral supernatant in 8 μg/mL of Polybrene transduction reagent (Merck Millipore, USA), and cells were then subjected to 48-hour post-transduction antibiotic selection using hygromycin at a final concentration of 1500 µg/mL (Nacalai Tesque, Japan). The positively transduced cells were subjected to serial dilutions to generate single cell-derived clones for each of HT29 and DLD1.

### 5. Protein extraction and western blotting

Upon trypsinisation and washing with 1x ice-cold phosphate-buffered saline, cells were lysed on ice for 30 min in 1X RIPA buffer (Nacalai Tesque, Japan) containing 1% SDS and 1: 100 protease inhibitor cocktails (Nacalai Tesque, Japan). The protein lysate was quantified using the Pierce™ BCA Protein Assay Kit (Thermo Scientific, USA) according to the manufacturer’s protocol. Proteins of equal mass (30 µg) were separated using 10% SDS-PAGE, transferred onto a nitrocellulose membrane (GE Healthcare Amersham), and blocked for one hour in blocking buffer; 5% non-fat dry milk prepared in 0.1% TBS-Tween 20 (TBS-T). The membrane was incubated overnight at 4 °C with a primary antibody, washed with 0.1% TBS-T three times on the next day, followed by one-hour secondary antibody incubation at room temperature. The membrane was washed three times with 0.1% TBS-T and signals were developed using a chemiluminescent substrate (SuperSignal™ West Pico PLUS, Thermo Scientific, USA), and visualized using the gel system (Gel Imaging System, Bio-Rad, USA). The primary antibodies of ENT2 (Santa Cruz, USA, 1: 1000) and GAPDH (Thermo Scientific, USA, 1: 1000) were diluted in blocking buffer. The secondary antibody comprised polyclonal rabbit anti-mouse IgG/HRP (Dako, Denmark, 1: 2000). The blots were visualised using Gel Doc XR Imaging System (BioRad). (S4 Fig).

### 6. PCR amplification and sanger sequencing

The *ENT2* region of interest was amplified from the genomic DNA of the single cell-derived clones. Briefly, 50 ng of the genomic DNA was amplified in a master mix consisting of 10 μM of each *ENT2* PCR forward and reverse primers, 0.25x polymerase (AmpliTaq Gold™ DNA Polymerase), PCR Buffer II, MgCl_2_, and dNTPs mix according to the manufacturer’s recommendations (Applied Biosystems). PCR amplification began by pre-incubating the mixture at 95 °C for 10 min, then by 35 repeated cycles of 95 °C for 15 sec annealing at 59.4 °C for 1 min, and extension at 72 °C for 50 sec using a thermal cycler (BioRad, USA). The PCR products were electrophoresed on a 1.5% agarose gel in 1X TAE buffer with a standard size marker of 100-bp DNA ladder (GeneRuler™, Thermo Scientific, USA). The gel was visualised using a gel-imaging system (Gel Doc™ XR-Imaging System, BioRad, USA). The PCR amplicons were column purified and subjected to Sanger sequencing using a 10 μM *ENT2* PCR forward primer (S5 Fig).

### 7. Cell viability

The viability of HT29/KO and DLD1/KO cell lines was determined using the cell proliferation assay (CellTiter 96® Non-Radioactive, Promega, USA). Cells at a density of 4 × 10^4^/well were seeded in a 96-well plate and incubated overnight at 37 °C and 5% CO_2_. The medium was gently removed and replaced with a fresh one. Each well was added with 15 μl of MTT dye solution (3-(4, 5-dimethylthiazol-2-yl)-2, 5-diphenyltetrazolium bromide) and incubated at 37 °C for four hours in the dark. Then, 100 µl of the solubilisation solution was added to each well and incubated for an hour at 37 °C. The absorbance was measured at 595 nm in a plate reader (VICTOR Multilabel, Perkin Elmer).

### 8. The colony-formation assay (CFA)

CFA was performed on a six-well plate. Each well was plated with 500 cells and incubated at 37 °C with 5% CO_2_. The medium was replaced every three days. On day 14, the medium was removed and cells were washed twice with 1X PBS. Fixation and staining were conducted by adding 1 mL of a mixture of 1% methyl violet stain (Fisher Scientific, USA) and 50% methanol and incubating at room temperature for 30 min (Buch et al., 2012). The stained colonies were rinsed with distilled water and left to dry at room temperature. Colonies were photographed using an imager (Doc XR Imager, BioRad, USA) and counted using the software Image J (http://imagej.net/Fiji/Downloads).

### 9. Bromodeoxyuridine/5-bromo-2'-deoxyuridine (BrdU) detection assay

The BrdU measurement was performed on a 96-well plate using the cell proliferation ELISA kit (Abcam, UK) according to the manufacturer’s protocol. Briefly, after overnight incubation of cells (2 x 10^4^/well) with the BrdU reagent (10 μl/well) at 37 °C, the medium was removed by tapping. Cells were then fixed and incubated at room temperature for 30 min. After removing the whole fixing solution by tapping, it was washed three times using 1X wash buffer (200 μl/well). Each well was then added with 100 μl of 1X peroxidase goat anti-mouse IgG conjugated antibody and incubated for 30 min at room temperature. Cells were rewashed three times before each well was added with 100 μl 3, 3', 5, 5'-Tetramethylbenzidine peroxidase substrate, and incubated for 30 min at room temperature. Finally, 100 μl stop solution was added to each well, and the absorbance was measured immediately at 450 nm.

### 10. Measurements of HPX and XO

The concentrations of HPX and XO were measured according to the manufacturer’s instructions for Amplex® Red HPX/XO assay (Invitrogen, USA). Briefly, cells were lysed using the assay kit buffer at an ice-cold temperature. The HPX concentration was measured by adding an appropriate amount of cell lysates with a 50 μL working solution containing 100 μM Amplex Red, 0.4 U/mL HRP, and 40 mU/mL XO. The XO activity was also measured by adding an appropriate amount of cell lysates with a 50 μL working solution containing 100 μM Amplex Red, 0.4 U/mL HRP, and 200 μM xanthine. The reactions were protected from light and incubated for 30 min at 37 °C. The absorbance was measured at 560 nm using a microplate reader (Infinite M200 PRO, Tecan).

### 11. Estimation of ROS production

The ROS production was estimated using the 2, 7-dichlorofluorescein diacetate (DCFDA) cellular ROS detection assay kit (Abcam, UK). According to the manufacturer’s protocol, each well was seeded with 2.5 x 10^4^ cells in a black 96-well plate and incubated overnight in a CO_2_ incubator at 37 °C. After removing the media, cells were washed using 100 µL of 1X buffer, stained with 25 µM DCFDA solution (100 μL/well), and incubated at 37 °C in the dark for 45 min. The DCFDA solution was removed by aspiration, and each well was added with 100 μL of 1X buffer. The fluorescence intensity was measured immediately using a fluorescence microplate reader (Infinite M200 PRO, Tecan) at Ex/Em = 485/535 nm.

### 12. Measurement of mitochondrial membrane potential (MMP)

MMP (ΔΨΜ) of HT29/KO and DLD1/KO cells were measured using a JC-1 MMP assay kit (Cayman, UK) according to the manufacturer’s protocol. Briefly, 4 x 10^4^ cells were plated into a black 96-well plate and incubated overnight at 37 °C. The JC-1 working staining solution (10 µL) was added to each well. The plates were incubated in a CO_2_ incubator for 20 min at 37 °C in the dark and then centrifuged for 5 min at 400 x g at room temperature. After removing the supernatant by gentle aspiration, cells were washed twice by adding 200 μl assay buffer to each well and centrifuged for 5 min at 400 x g at room temperature. Finally, 100 μl assay buffer was added to each well, and the fluorescence intensity of cells was quantified at Ex/Em = 535/595 nm for J-aggregates (red) and Ex/Em = 485/535 nm for monomers (green). Also, the fluorescent stain of cells was detected qualitatively using an inverted fluorescence microscope (Olympus, Japan) with Ex/Em = 540/570 nm filter for JC-1 J-aggregates and Ex/Em = 485/535 nm filter for JC-1 monomers.

### 13. Statistical analysis

All data were statistically analysed using the SPSS software (version 28, IBM, International Business Machines Corp, New York). The results were expressed as mean ± SD obtained from three independent experiments where each assay was performed in triplicates (n = 9) at the significance level of 0.05 (P ≤ 0.05). Data normality was assessed by Shapiro–Wilk normality test. For qRT-PCR analysis, the relative normalised *ENT2* expression was determined using the 2^-∆∆Ct^ method followed by the one-way analysis of variance (ANOVA) to determine if the experimental groups of normal colon cell line and CRC cell lines were different. Also, the experimental groups of HT29/KO were evaluated if they were different using the parametric one-way ANOVA. Besides, the independent t-test was performed to determine two experimental groups of DLD1/KO were different. Finally, the concentrations of HPX and XO for both experimental groups of HT29/KO and DLD1/KO were evaluated using the non-parametric Kruskal Wallis test and Mann-Whitney U test, respectively.

## Results

### 1. Higher *ENT2* expression in CRC cell lines than the normal colon cell line

**Fig 1:**
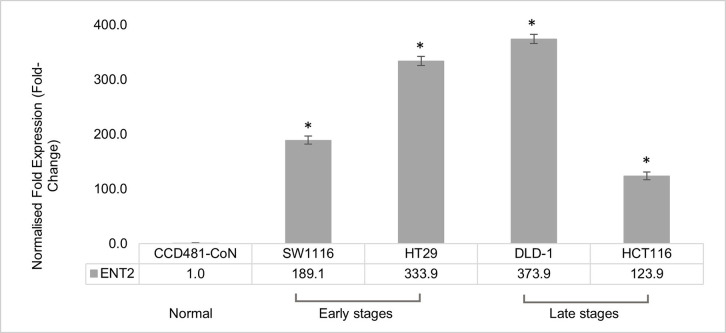
Normalised expression of *ENT2* in CRC cell lines, i.e., SW1116 (Dukes’ A), HT29 (Dukes’ B), DLD1 (Dukes’ C), and HCT116 (Dukes’ D) against the differential normalised expression of the *ENT2* gene in normal colon epithelial cell Lines CCD-841CoN. *GAPDH* and *HPRT1* were the reference genes. Data are presented as means ± SD. * indicates a significant difference between normal and CRC cell lines (P ≤ 0.05). Statistical differences were examined using the one-way ANOVA test.

A panel of CRC cell lines, representing early and late stages of CRC and normal colon cell line were subjected to qRT-PCR analysis to determine the expression level of *ENT2* gene. Fig 1 shows a significantly higher (P ≤ 0.05) *ENT2* expression in CRC cell lines than in the normal colon cells via the qRT-PCR analysis. The *ENT2* expression in SW116 (Dukes’ A), HT29 (Dukes’ B), DLD1 (Dukes’ C), and HCT116 (Dukes’ D) CRC cells was 189, 333, 373, and 123-fold higher than the CCD841CoN normal colon cells, respectively.

### 2. Transduction and assessment of the editing efficiency by CRISPR/Cas9

Western blotting of ENT2-targeted CRC cells pool (HT29 and DLD1) showed that the sgENT2_4 construct had the best targeting efficiency among the tested five sgENT2 constructs. The study proceeded with isolating single cell from the sgENT2-4/ KO cells pool to find the single cell-derived clones that had the best ENT2 protein reduction.

**Fig 2:**
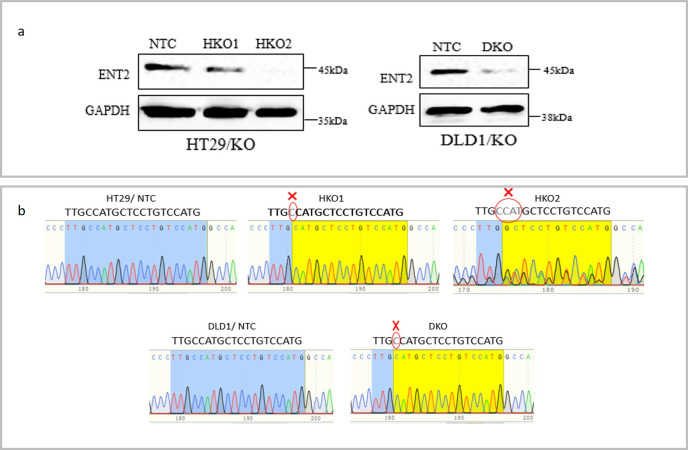
Assessment of the editing efficiency. (A) Western blotting of clones with positive edit for both HT29/KO and DLD1/KO. The Immunoblots show the ENT2 expression status in the cells. The GAPDH protein expression was used as a loading control. (B) Sanger sequencing of HT29/KO and DLD1/KO clones. The chromatogram of the control clone (NTC) of both HT29 and DLD1 cells shows an unedited sequence for the sgENT2-4 target region. The chromatogram of HKO1 showed a single-base deletion of cytosine (C), while HKO2 showed a messy four base pair deletion (CCAT). The chromatogram of DKO shows a single-base deletion of cytosine (C).

Fifty-three single cell-derived clones of both HT29/KO and DLD1/KO cells and their respective non-targeting construct (NTC) clones were screened via Western blotting to detect the ENT2 expression level (data not shown). Western blotting analysis of *ENT2* knockout of HT29 (HT29/KO) and DLD1 (DLD1/KO) single cell-derived clones showed several single cell-derived clones with complete and partial *ENT2* knockout compared to their respective NTC clones. For HT29/KO, there were two clones of *ENT2* KO HT29 (HT29/KO) single cell-derived clones with *ENT2* partially KO (HKO1) and complete *ENT2* KO (HKO2), and one clone of DLD1/KO single cell-derived clone (DKO) with almost *ENT2* KO (Fig 2A).

Sanger sequencing analysis of HKO1, HKO2 and DKO revealed frameshift mutation in these clones due to the base(s) deletion. These clones (HKO1, HKO2, and DKO) and their respective NTC clones were used in the subsequent functional assays (Fig 2B).

### 3. The *ENT2* knockout induced purine catabolic pathway

**Fig 3:**
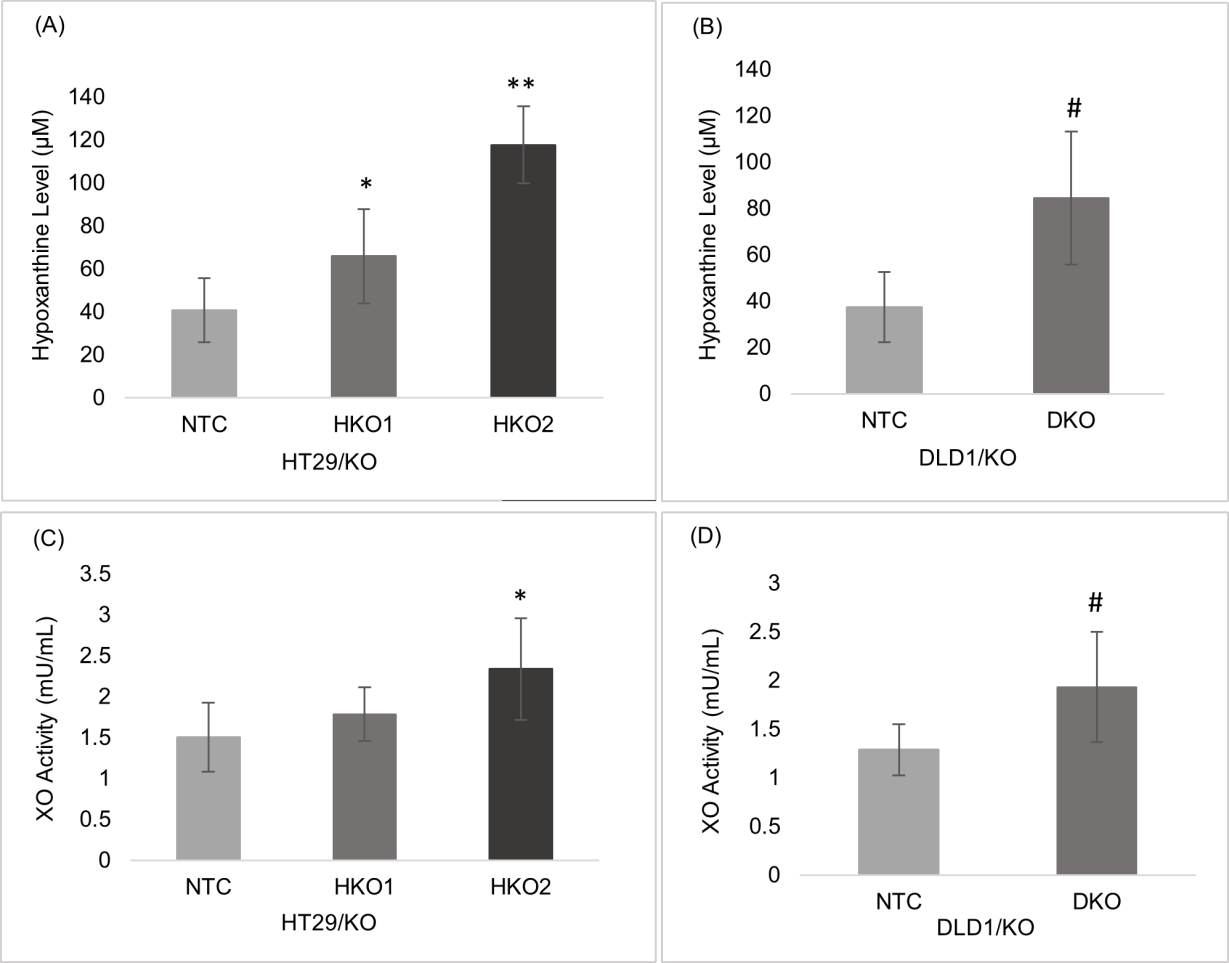
The HPX level and XO activity HT29/KO and DLD1/KO Cells. (A) HPX level of HKO1 and HKO2 was significantly higher than the NTC control with 1.6- and 2.9-fold higher, respectively. (B) HPX level of DKO was significantly higher with 2.2-fold than the NTC control. (C) XO activity of HKO2 was significantly higher than the NTC control with 1.5-fold. (D) XO activity of DKO was significantly higher than the NTC control with 1.5-fold. Data are presented as means ± SD. * indicates a significant difference between NTC vs. HKO1 or HKO2 cell; *P ≤ 0.001, ** P ≤ 0.001, while # indicates significant difference between NTC vs. DKO.

HPX is the first metabolite in the purine catabolism pathway, which metabolised into xanthine and uric acid and superoxide by XO enzyme. In this study, the HPX concentration in HT29/KO cells increased significantly (P ≤ 0.05, P ≤ 0.001) in HKO1 and HKO2 with 1.6 and 2.9-fold higher, respectively, than in the NTC control cells. Also, the HPX level was 1.7-fold higher in HKO2 than in HKO1 cells (Fig 3A). Likewise, the HPX level in DKO cells was significantly higher (P ≤ 0.05) than in the NTC control cells of DLD1/KO by 2.2-fold (Fig 3B). The XO activity of HT29/KO cells increased significantly in HKO2 clones (P ≤ 0.05). It was 1.5-fold higher than the NTC control cells (Fig 3C). Likewise, the XO activity was significantly higher (P ≤ 0.05), i.e., 1.5-fold higher in DKO cells than in the NTC control cells of DLD1/KO (Fig 3D).

### 4. The *ENT2* knockout increased the production of ROS

**Fig 4:**
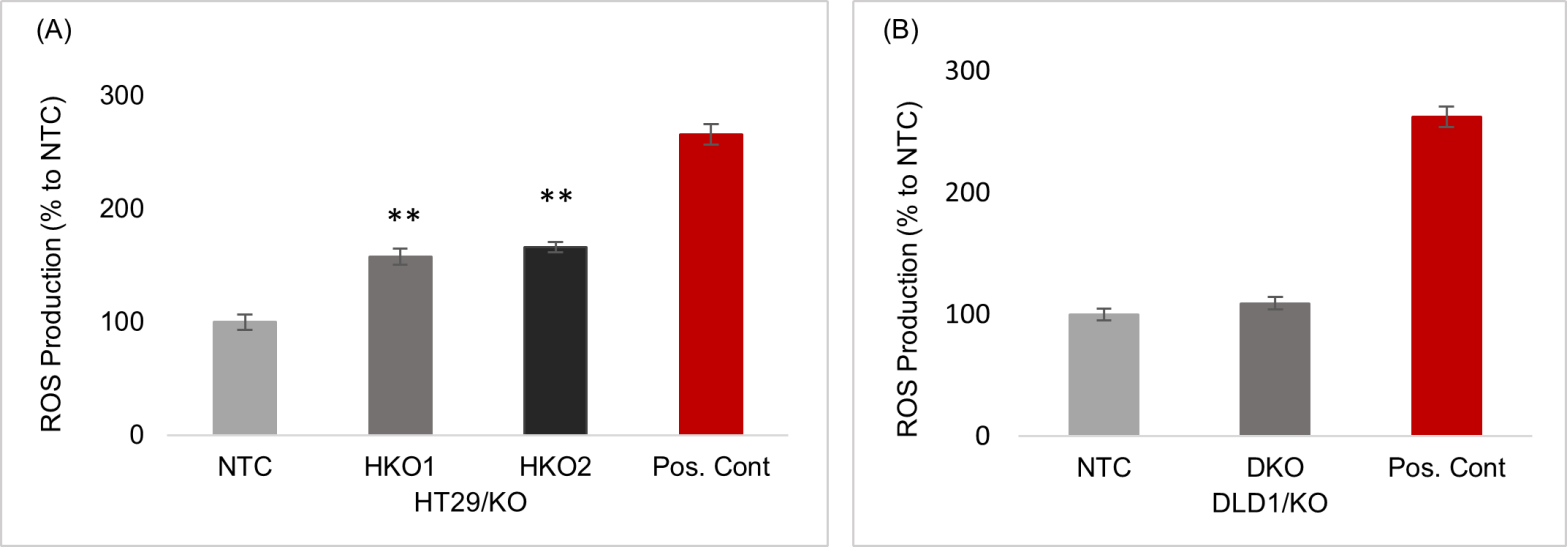
The production of ROS in *ENT2* knockout cells. (A) The ROS level in HT29/KO cells for both HKO1 and HKO2 was significantly higher than NTC. (B) The ROS level in DLD1/KO cells showed no difference between DKO and NTC cells. Data are presented as means ± SD and expressed as % relative to the control NTC. ** P ≤ 0.001. Positive control is tert-Butyl Hydroperoxide (TBHP).

The intercellular ROS was quantitatively measured using the cell-permeable probe (DCFDA). Fig 4A shows the results of ROS production. It was significantly higher (P ≤ 0.001) in HT29/KO clones (HKO1 and HKO2) than in the NTC control. By contrast, the DKO of DLD1/KO cells was not significantly different from the NTC control (Fig 4B).

### 5. The *ENT2* knockout affected the cell proliferation and survival of the early stages of CRC cell lines

**Fig 5:**
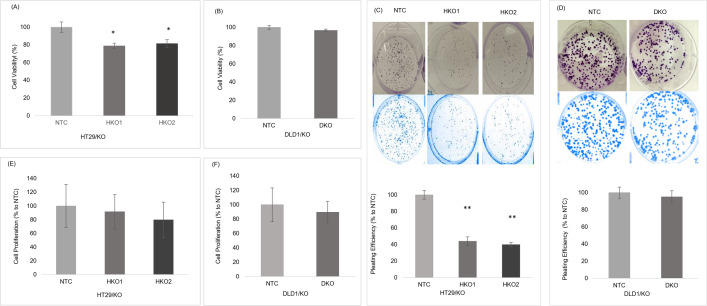
The *ENT2* knockout effects on CRC proliferation and survival. (A) Cell viability of HT29/KO as measured by the MTT assay. It was significantly lower in both HKO1 and HKO2 than in the control NTC (B) Cell viability of DLD1/KO with no significant difference between the DKO clone and NTC cells. (C) Colonies of HT29/KO, taken using Doc XR Imager, BioRad. (D) Colonies of DLD1KO. Each well of a six-well plate was plated with 500 cells and incubated for 14 days. Plating Efficiency (PE) was calculated as the ratio of the number of colonies to the number of cells seeded. PE decreased significantly in both HKO1 and HKO2. No significant difference occurred between NTC and DKO cells. (E) Cell proliferation of HT29/KO cells as measured by the BrdU cell proliferation ELISA assay. (F) Cell proliferation of DLD1/KO cells. Data are presented as means ± SD and expressed as % relative to control cells. * indicates a significant difference between NTC vs. KO1 or KO2; *P ≤ 0.05, **P ≤ 0.001.

Cell viability of HT29/KO and DLD1/KO was the first approach used in this study to evaluate the effect of ENT2 knockout on cell proliferation. MTT assay was performed the cellular metabolic activity as an indicator of cell viability. Fig 5A and 5B show that the cell viability of HT29/KO clones (HKO1 and HKO2) was significantly lower (P ≤ 0.05) than the NTC control cells. By contrast, the DLD1/KO clone (DKO) was not different from the NTC control. Cell survival based on the ability of single cells to grow into colonies was the second approach to determine cell proliferation. Fig 5C and 5D show that the colony formation of CRC cells in HT29/KO clones (HKO1 and HKO2) was significantly lower (P ≤ 0.001) than in the NTC control. No difference occurred between the DLD1/KO clone (DKO) and the NTC controls. The third approach used to evaluate the effect of ENT2 knockout on cell proliferation was detecting BrdU incorporation into newly synthesised DNA of actively proliferating cells. In this assay, *ENT2* knockout impaired the newly synthesised DNA in both HT29/KO and DLD1/KO cells compared to their controls with no significant difference (Fig 5E and 5F).

### 6. *ENT2* knockout increased the apoptosis of early stages CRC cell lines

**Fig 6:**
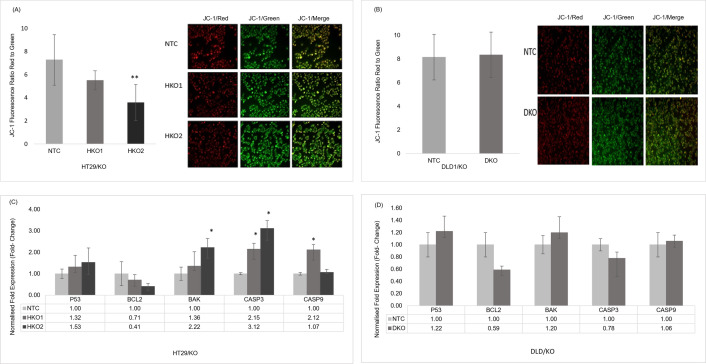
The fluorescent intensity of cells with JC-1 stains: (A) HT29/KO and (B) DLD1/KO. Quantitative data (histograms) are expressed in the ratio of JC-1 fluorescence red/green. Photographic data show JC-1 red (J-aggregate), JC-1 green (J-monomer), and the merged red-green image. The red/green fluorescence intensity was detected using a fluorescence microscope at the scale bar of 100 μm. (C) Expression profiling of some apoptosis-associated genes in HT29/KO. (D) Expression profiling of some apoptosis-associated genes in DLD1/KO. *GAPDH* and *HPRT1* served as the reference genes. Data are presented as means ± SD. * indicates a significant difference between NTC vs. CRC clone cells. *P ≤ 0.05, **P ≤ 0.001.

Apoptosis indication in this study was evaluated through the integrity of the mitochondrial membrane examination and gene expression profiling of some apoptosis-associated genes. Fig 6A shows the loss of MPP (measured in ΔΨM) in HT29/KO, i.e., the red/green ratio was significantly lower (P ≤ 0.001) in the HKO2 clone than in the NTC clone cells. No significant difference occurred between the DKO clone of DLD1/KO cells and the NTC control (Fig 6B).

The gene expression profiling of apoptosis-associated genes, such as tumour suppressor gene, anti-apoptotic *P53* gene, pro-apoptotic *BCL2*, *BAK*, and Caspases (*Caspase-3* and *Caspase-9*), was assessed by quantitative real-time PCR for HT29/KO and DLD1/KO cells. Meanwhile, Fig 6C and 6D show that *P53, BCL2, BAK, Caspase-3*, and *Caspase-9* expressed differently at the regulation level, indicating the apoptosis pathway was activated in both HT29/KO and DLD1/KO cells.

## Discussion

Cancer cells require high nucleotide turnover, often achieved through upregulation of equilibrative nucleoside transporters (ENTs) to support DNA synthesis [21–23]. In this study, *ENT2* gene expression showed significant upregulation in all CRC stages cell lines compared to the normal colon cell line. This result agreed with our previous study [18] and the findings of other studies [15–17].

Tumour cells intensified the purine salvage pathway to accelerate their growth and proliferation [24] by shifting HPX from the catabolic to the salvage pathway via hypoxanthine-guanine phosphoribosyltransferase (HPRT) enzyme [11] to meet the demand for nucleic acid synthesis [25]. Thus, the downregulation of *ENT2* expression could be deleterious to the cells due to interruption in the HPX efflux, which, in turn, promoted the purine catalysis to generate ROS as a by-product.

“As observed in both HT29/KO and DLD1/KO, ENT2 knockout led to HPX accumulation, thereby increasing XO activity and downstream ROS production, as corroborated by similar mechanisms in other cancers [26,27].”

In this study, the significant increase in HPX level in all clones of both HT29/KO and DLD1/KO agreed with the result of another study, suggesting that ENT2 was the main transporter responsible for the HPX efflux of the cell [26]. Incidentally, HPX level was elevated in CRC cells [24], and this increment was associated with the advanced CRC stages [22,28]. In this study, the *ENT2* knockout was effectively accelerated the purine catabolic pathway by activating the HPX catabolism which in turn, increased the XO activity significantly in both HT29/KO and DLD1/KO clones. These findings agreed with the finding of a recent study [29] where the authors found that a high HPX level increased the XO activity in human liver cancer cells.

Meanwhile, the increase in XO activity is generally associated with ROS elevation [27]. The data of the early stage of CRC cell line, HT29/KO, confirmed this phenomenon, i.e., increased XO activity was concurrent with the significant elevation of ROS level by 1.58-fold in the partial ENT2 knockout clone (HKO1) and 1.66-fold in the complete ENT2 knockout clone (HKO2). Thus, the increase in the XO activity was probably a consequence of increasing HPX level and closely related to the higher ROS level in the early stages of CRC cells. Interestingly, in the late CRC stage cell line, the XO activity of DLD1/KO increased but with a weak elevation of the ROS level, i.e., 1.2-fold only. This low level of ROS might be due to the antioxidant properties of uric acid, the end product of the purine catabolism pathway. Uric acid contributes 60% of the free radical scavenging activity [30] as it neutralizes the hydroxyl radicals [31], prevents the degradation of the superoxide dismutase enzyme, and removes the superoxide [30,32]. Thus, impaired HPX transport disrupts the redox balance in CRC cells.

The expression of ENT2 is mostly located at the cell membrane [10]. In this study, the *ENT2* knockout strongly impacted the viability of HT29/KO cells. Similarly, the colony formation data, i.e., a common metastasis indicator that measures the tumour spreading capacity [33], showed that the *ENT2* knockout significantly inhibited the HT29/KO tumorigenesis in vitro. This finding agreed with the result of another study on dipyridamole that acted as a selective inhibitor for ENT transporters [34], thereby reducing tumour growth of the primary triple-negative breast cancer cells and metastasis [35] (S3–S4 Table).

Our BrdU assay showed that the *ENT2* knockout hampered DNA synthesis in both clones of HT29/KO cells, eventually affecting cell proliferation. A decrease in DNA synthesis might be directly related to the lack of endogenous nucleoside transportation due to the blocking of *ENT2* variants in the nuclear membrane. This finding was consistent with the result of another study, in which the knockdown of *ENT2* isoforms led to a striking decrease in cell proliferation and a dysregulation of the cell cycle, causing the arrest of cells in the S phase and a reduction of cells in the G2 phase due to insufficient nucleotide pools for DNA synthesis [7].

Besides, an insufficient supply of nucleotides during DNA replication caused a slow replication, eventually damaging the DNA with a low rate of cell proliferation [36]. In this study, the *ENT2* knockout showed little impact on cell viability with weak inhibition on tumorigenesis and DNA synthesis in the cell line of the late CRC stage cell line (DLD1/KO). This mild effect might be due to the modification of the metabolic pathway or modulation of gene expression. These modulations are somewhat common in cancer cells, especially in advanced stages, to adapt and thrive in a harsh environment [24]. The HPX concentration generally affected the expression of *ENT* family genes to maintain cell homeostasis [26]. The increment of HPX level up-regulated the *ENT* genes in the CRC cell line Caco-2 [26]. Although ENT2 transports HPX readily, ENT1 shows a low affinity for the transportation of HPX [37]. In this study, in the absence or disruption of *ENT2*, cells might resort to a compensatory upregulation of other closely related ENT transporters, notably ENT1, to prevent damage from poor HPX translocation [37]. However, these findings need further investigation.

In determining the role of *ENT2*, this study speculated that the *ENT2* knockout might induce the intrinsic pathway of apoptosis in response to ROS elevation following the modulation of the purine catabolism pathway. In this respect, changes in MMP as an indication of mitochondrial dysfunction were detected in HT29/KO cell line. Both clones of HT29/KO showed a loss of MMP (ΔΨm) with a higher capacity in HKO2 than HKO1. This finding was consistent with the result of another study, i.e., the XO-mediated ROS generation was involved in mitochondrial dysfunction, reducing cell viability [38].

Several studies have shown that inhibiting XO prevented ROS accumulation, and hence, avoided the detrimental effect of ROS generation [39] such as mitochondrial dysfunction [40,41]. Unlike the HT29/KO cells, the elevation of ROS level was low in DLD1/KO cells, and hence, showed no changes in MPP compared to NTC cells. This result suggested that a small increment in the ROS level might not be sufficiently cytotoxic to induce apoptosis. While the reduction of MMP might serve as an early sign of apoptosis [42,43]. Therefore, the loss of MMP in this study might indicate that the apoptosis mechanism had been initiated [38]. For further confirmation of this possibility, the role of some genes in *ENT2* knockout-induced apoptosis was investigated in HT29/KO and DLD1/KO cells.

In this study, HT29/KO showed a decrease in the expression of *BCL2* but with an increment in *BAK* and *P53* expression in both partial and complete *ENT2* knockout clones. This differential expression suggested that the induction of apoptosis had started. This finding was consistent with the result of another study, suggesting that the increased ROS levels elicited tumour cell death by damaging the mitochondria while promoting apoptosis via disrupting the equilibrium between the anti- and pro-apoptotic genes [44]. The expression of *Caspase-3* and *Caspase-9* was upregulated in both HKO1 and HKO2 cells of HT29/KO with significant up-regulation in *Caspase-3* compared to NTC cells While *Caspase-9* was significantly upregulated in HKO1 only. Notably, *Caspase-3* showed higher activity than *Caspase-9* of HKO2 cells. *Caspase-3* is responsible for multiple cellular protein degradation and hence, indispensable for DNA fragmentation in apoptosis [45].

On the other hand, due to the small increment in the ROS level, DLD1/KO clone cells did not show significant differences in the apoptotic-associated gene expression. In this study, the upregulation of gene expression of *P53* and *BAK* and a reduction in the *BCL2* gene expression did not relate to an increment in the expression of *Caspase-3* and *Caspase-9* expressions in the late stages of the CRC cell line. Although the increment in the *P53* expression might, in principle, indicate the cells would enter the initial phase of cell death and apoptosis, it might also activate the antioxidant pathways by the upregulation of the antioxidant genes. A study reported that *P53* had a dual opposed role in controlling the ROS levels by either promoting the oxidant or the antioxidant gene expression [46]. Interestingly, the expression of *Caspase-3* showed a decrease in its mRNA expression. *Caspase-3* is often inactivated in cancer cells, causing cells resisting to microenvironmental stresses and chemotherapy treatments [45,47,48]. This finding might indicate the resistance that appeared in DLD1/KO to oxidative stress resulting from the induction of the purine catabolic pathway.

## Conclusion

Given that the relationship between ENT2 gene expression and purine catabolism remains poorly explored, this study investigates the connection between ENT2 and apoptosis by promoting the purine catabolism pathway in both early and late-stage CRC cell lines. The findings suggested that the elevation of ROS production resulting from the purine catabolic pathway induced the intrinsic apoptosis pathway, particularly in the early stage of the CRC cell line. The somewhat opposing results between the early and late CRC stages cell lines may be attributed to the difference in cancer progression since cancer is generally more resistant and well-adapted to thrive in a harsh environment in advanced stages. Additionally, biological variation and the mutational status of cell lines may contribute to these opposing differences. Overall, the strategy for inducing apoptosis may be more complex than initially anticipated. To better understand the ENT2-mediated apoptosis pathway, further investigation into mechanisms such as apoptotic signaling, TNF, Fas-L, and TRAIL pathways is essential. These pathways have well-established roles in apoptosis and potential interactions with oxidative stress, a significant byproduct of purine catabolism. The TNF and Fas-L pathways are recognized extrinsic apoptotic mechanisms that can be activated by cellular stress and may contribute to ENT2-mediated apoptosis in CRC cells. Similarly, the TRAIL pathway selectively induces apoptosis in cancer cells and is modulated by ROS production. Since our findings suggest intrinsic apoptosis activation in response to elevated ROS levels, exploring the involvement of extrinsic apoptotic pathways, such as TNF and Fas-L, will offer a more comprehensive understanding of ENT2’s role in apoptosis.

To our knowledge, this is the first study to practically investigate the relationship between ENT2 and apoptosis by promoting the purine catabolism pathway across both early and late-stage CRC cell lines. However, this study has some limitations. Instead of pooled cells, this study used the *ENT2* knockout clonal cells with a limited number of clones. Comparing more than two clones for each cell line and including other cell lines would be more viable. Evaluating ENT1 expression, HPRT, and uric acid levels, along with in vivo models and patient-derived organoid systems, could help further validate the translational relevance of these findings. Exploring the potential activation of compensatory transporters or alternative mechanisms is also an important avenue to investigate. Future studies may benefit from transcriptomic and proteomic analyses to better clarify ENT2-related apoptotic signaling pathways.

Despite these limitations, this study enhances our understanding of the role of *ENT2* in inducing apoptosis cell death via the purine catabolic pathway in CRC. It could also pave the way for exploring the potential role of ENT2 as a promising strategy for CRC treatment by impairing DNA synthesis and inhibiting cell growth.

## Supporting information

S1 Table Primer list. (PDF)

S2 Table (a and b) The sgRNAs sequences targeting the ENT2 gene and the schematic diagram of sgENT2s designs and their target sequence location relative to the entire ENT2 gene structure. (ZIP)

S3 Fig Sanger sequencing verification for the ligated sgENT2 constructs; sgENT2-1 to 5. (PDF)

S4 Fig Western blots of the positive edit clones of both HT29/KO and DLD1/KO. (PDF)

S5 Fig PCR forward and reverse primers flanking the sgENT2-4 genomic target site. (PDF)

## References

[1] Bray F, Laversanne M, Sung H, Ferlay J, Siegel RL, Soerjomataram I, et al. Global cancer statistics 2022: GLOBOCAN estimates of incidence and mortality worldwide for 36 cancers in 185 countries. CA Cancer J Clin. 2024;74(3):229–63. doi: 10.3322/caac.21834 38572751

[2] Xi Y, Xu P. Global colorectal cancer burden in 2020 and projections to 2040. Transl Oncol. 2021;14(10):101174. doi: 10.1016/j.tranon.2021.101174 34243011 PMC8273208

[3] Arnold M, Sierra MS, Laversanne M, Soerjomataram I, Jemal A, Bray F. Global patterns and trends in colorectal cancer incidence and mortality. Gut. 2017;66(4):683–91. doi: 10.1136/gutjnl-2015-310912 26818619

[4] Wiseman MJ. Nutrition and cancer: prevention and survival. Br J Nutr. 2019;122(5):481–7. doi: 10.1017/S0007114518002222 30213279

[5] Matsuyama M, Wakui M, Monnai M, Mizushima T, Nishime C, Kawai K, et al. Reduced CD73 expression and its association with altered purine nucleotide metabolism in colorectal cancer cells robustly causing liver metastases. Oncol Lett. 2010;1(3):431–6. doi: 10.3892/ol_00000076 22966321 PMC3436221

[6] Zhu J, Djukovic D, Deng L, Gu H, Himmati F, Chiorean EG, et al. Colorectal cancer detection using targeted serum metabolic profiling. J Proteome Res. 2014;13(9):4120–30. doi: 10.1021/pr500494u 25126899

[7] Grañé-Boladeras N, Spring CM, Hanna WJB, Pastor-Anglada M, Coe IR. Novel nuclear hENT2 isoforms regulate cell cycle progression via controlling nucleoside transport and nuclear reservoir. Cell Mol Life Sci. 2016;73(23):4559–75. doi: 10.1007/s00018-016-2288-9 27271752 PMC11108336

[8] Pedley AM, Benkovic SJ. A new view into the regulation of purine metabolism: the purinosome. Trends Biochem Sci. 2017;42(2):141–54. doi: 10.1016/j.tibs.2016.09.009 28029518 PMC5272809

[9] Boswell-Casteel RC, Hays FA. Equilibrative nucleoside transporters-a review. Nucleosides Nucleotides Nucleic Acids. 2017;36(1):7–30. doi: 10.1080/15257770.2016.1210805 27759477 PMC5728162

[10] Young JD, Yao SYM, Baldwin JM, Cass CE, Baldwin SA. The human concentrative and equilibrative nucleoside transporter families, SLC28 and SLC29. Mol Aspects Med. 2013;34(2–3):529–47. doi: 10.1016/j.mam.2012.05.007 23506887

[11] Garcia-Gil M, Camici M, Allegrini S, Pesi R, Petrotto E, Tozzi MG. Emerging role of purine metabolizing enzymes in brain function and tumors. Int J Mol Sci. 2018;19(11):3598. doi: 10.3390/ijms19113598 30441833 PMC6274932

[12] Lu H, Li X, Lu Y, Qiu S, Fan Z. ASCT2 (SLC1A5) is an EGFR-associated protein that can be co-targeted by cetuximab to sensitize cancer cells to ROS-induced apoptosis. Cancer Lett. 2016;381(1):23–30. doi: 10.1016/j.canlet.2016.07.020 27450723 PMC5017913

[13] Antonioli L, Blandizzi C, Pacher P, Haskó G. Immunity, inflammation and cancer: a leading role for adenosine. Nat Rev Cancer. 2013;13(12):842–57. doi: 10.1038/nrc3613 24226193

[14] Naes SM, Ab-Rahim S, Mazlan M, Abdul Rahman A. equilibrative nucleoside transporter 2: properties and physiological roles. BioMed Res Int. 2020;2020:5197626. doi: 10.1155/2020/5197626 33344638 PMC7732376

[15] Al-Abdulla R, Perez-Silva L, Abete L, Romero MR, Briz O, Marin JJG. Unraveling “The Cancer Genome Atlas” information on the role of SLC transporters in anticancer drug uptake. Expert Rev Clin Pharmacol. 2019;12(4):329–41. doi: 10.1080/17512433.2019.1581605 30744443

[16] Mukhopadhya I, Murray GI, Berry S, Thomson J, Frank B, Gwozdz G, et al. Drug transporter gene expression in human colorectal tissue and cell lines: modulation with antiretrovirals for microbicide optimization. J Antimicrob Chemother. 2016;71(2):372–86. doi: 10.1093/jac/dkv335 26514157

[17] Liu Y, Zuo T, Zhu X, Ahuja N, Fu T. Differential expression of hENT1 and hENT2 in colon cancer cell lines. Genet Mol Res. 2017;16(1). doi: 10.4238/gmr16019549 28218790

[18] Naes SM, Ab-Rahim S, Mazlan M, Amir Hashim NA, Abdul Rahman A. Increased ENT2 expression and its association with altered purine metabolism in cell lines derived from different stages of colorectal cancer. Exp Ther Med. 2023;25(5):212. doi: 10.3892/etm.2023.11911 37123217 PMC10133795

[19] Schmittgen TD, Livak KJ. Analyzing real-time PCR data by the comparative C(T) method. Nat Protoc. 2008;3(6):1101–8. doi: 10.1038/nprot.2008.73 18546601

[20] Syafruddin SE, Rodrigues P, Vojtasova E, Patel SA, Zaini MN, Burge J, et al. A KLF6-driven transcriptional network links lipid homeostasis and tumour growth in renal carcinoma. Nat Commun. 2019;10(1):1152. doi: 10.1038/s41467-019-09116-x 30858363 PMC6411998

[21] Noguchi S, Takagi A, Tanaka T, Takahashi Y, Pan X, Kibayashi Y, et al. Fluorouracil uptake in triple-negative breast cancer cells: Negligible contribution of equilibrative nucleoside transporters 1 and 2. Biopharm Drug Dispos. 2021;42(2–3):85–93. doi: 10.1002/bdd.2261 33426680

[22] Pastor-Anglada M, Pérez-Torras S. Emerging roles of nucleoside transporters. Front Pharmacol. 2018;9:606. doi: 10.3389/fphar.2018.00606 29928232 PMC5997781

[23] Pizzagalli MD, Bensimon A, Superti‐Furga G. A guide to plasma membrane solute carrier proteins. FEBS J. 2021;288(9):2784–835. doi: 10.1111/febs.15531 32810346 PMC8246967

[24] Ong ES, Zou L, Li S, Cheah PY, Eu KW. Metabolic profiling in colorectal cancer reveals signature metabolic shifts during tumorigenesis. Mol Cellul Proteom. 2010. doi: 10.1074/mcp.M900551-MCP200 20147338

[25] Townsend MH, Anderson MD, Weagel EG, Velazquez EJ, Weber KS, Robison RA, et al. Non-small-cell lung cancer cell lines A549 and NCI-H460 express hypoxanthine guanine phosphoribosyltransferase on the plasma membrane. Onco Targets Ther. 2017;10:1921–32. doi: 10.2147/OTT.S128416 28408844 PMC5384690

[26] Senyavina NV, Tonevitskaya SA. Effect of hypoxanthine on functional activity of nucleoside transporters ENT1 and ENT2 in Caco-2 polar epithelial intestinal cells. Bull Exp Biol Med. 2015;160(1):160–4. doi: 10.1007/s10517-015-3118-z 26593410

[27] Wang Y, Cui Q, Wang C, Liu S, Du R, Tian S. C1QBP regulates apoptosis of renal cell carcinoma via modulating xanthine dehydrogenase (XDH) mediated ROS generation. 2020. 10.7150/ijms.71703 PMC9149634 35693733

[28] Amir Hashim NA, Ab-Rahim S, Wan Ngah WZ, Nathan S, Ab Mutalib NS, Sagap I, et al. Global metabolomics profiling of colorectal cancer in Malaysian patients. Bioimpacts. 2021;11(1):33–43. doi: 10.34172/bi.2021.05 33469506 PMC7803921

[29] Toledo-Ibelles P, Gutiérrez-Vidal R, Calixto-Tlacomulco S, Delgado-Coello B, Mas-Oliva J. Hepatic accumulation of hypoxanthine: a link between hyperuricemia and nonalcoholic fatty liver disease. Arch Med Res. 2021;52(7):692–702. doi: 10.1016/j.arcmed.2021.04.005 33966916

[30] Yao JK, Dougherty GG, Reddy RD, Matson WR, Kaddurah-Daouk R, Keshavan MS. Associations between purine metabolites and monoamine neurotransmitters in first-episode psychosis. Front Cell Neurosci. 2013;7:90. doi: 10.3389/fncel.2013.00090 23781173 PMC3678099

[31] Davies KJ, Sevanian A, Muakkassah-Kelly SF, Hochstein P. Uric acid-iron ion complexes. A new aspect of the antioxidant functions of uric acid. Biochem J. 1986;235(3):747–54. doi: 10.1042/bj2350747 3753442 PMC1146751

[32] Ghanbari Movahed Z, Rastegari-Pouyani M, Mohammadi MH, Mansouri K. Cancer cells change their glucose metabolism to overcome increased ROS: One step from cancer cell to cancer stem cell? Biomed Pharmacother. 2019;112:108690. doi: 10.1016/j.biopha.2019.108690 30798124

[33] Alkan HF, Vesely PW, Hackl H, Foßelteder J, Schmidt DR, Vander Heiden MG, et al. Deficiency of malate-aspartate shuttle component SLC25A12 induces pulmonary metastasis. Cancer Metab. 2020;8(1):26. doi: 10.1186/s40170-020-00232-7 33292758 PMC7690131

[34] Cuthbertson CR, Guo H, Kyani A, Madak JT, Arabzada Z, Neamati N. The Dihydroorotate Dehydrogenase Inhibitor Brequinar Is Synergistic with ENT1/2 Inhibitors. ACS Pharmacol Transl Sci. 2020;3(6):1242–52. doi: 10.1021/acsptsci.0c00124 33344900 PMC7737209

[35] Spano D, Marshall J-C, Marino N, De Martino D, Romano A, Scoppettuolo MN, et al. Dipyridamole prevents triple-negative breast-cancer progression. Clin Exp Metastasis. 2013;30(1):47–68. doi: 10.1007/s10585-012-9506-0 22760522 PMC7654221

[36] Bester AC, Roniger M, Oren YS, Im MM, Sarni D, Chaoat M. Nucleotide deficiency promotes genomic instability in early stages of cancer development. Cell. 2011;145(3):435–46. doi: 10.1016/j.cell.2011.03.044 21529715 PMC3740329

[37] Altaweraqi RA, Yao SYM, Smith KM, Cass CE, Young JD. HPLC reveals novel features of nucleoside and nucleobase homeostasis, nucleoside metabolism and nucleoside transport. Biochim Biophys Acta Biomembr. 2020;1862(7):183247. doi: 10.1016/j.bbamem.2020.183247 32126230

[38] Pelin M, Fusco L, Martín C, Sosa S, Frontiñán-Rubio J, González-Domínguez JM, et al. Graphene and graphene oxide induce ROS production in human HaCaT skin keratinocytes: the role of xanthine oxidase and NADH dehydrogenase. Nanoscale. 2018;10(25):11820–30. doi: 10.1039/c8nr02933d 29920573

[39] Linder N, Martelin E, Lundin M, Louhimo J, Nordling S, Haglund C, et al. Xanthine oxidoreductase–clinical significance in colorectal cancer and in vitro expression of the protein in human colon cancer cells. Eur J Cancer. 2009;45(4):648–55. doi: 10.1016/j.ejca.2008.10.036 19112016

[40] Gladden JD, Zelickson BR, Wei C-C, Ulasova E, Zheng J, Ahmed MI, et al. Novel insights into interactions between mitochondria and xanthine oxidase in acute cardiac volume overload. Free Radic Biol Med. 2011;51(11):1975–84. doi: 10.1016/j.freeradbiomed.2011.08.022 21925594 PMC3364106

[41] Vergeade A, Mulder P, Vendeville C, Ventura-Clapier R, Thuillez C, Monteil C. Xanthine oxidase contributes to mitochondrial ROS generation in an experimental model of cocaine-induced diastolic dysfunction. J Cardiovasc Pharmacol. 2012;60(6):538–43. doi: 10.1097/FJC.0b013e318271223c 22967988

[42] Petit PX, Lecoeur H, Zorn E, Dauguet C, Mignotte B, Gougeon ML. Alterations in mitochondrial structure and function are early events of dexamethasone-induced thymocyte apoptosis. J Cell Biol. 1995;130(1):157–67. doi: 10.1083/jcb.130.1.157 7790370 PMC2120516

[43] Choi JB, Kim JH, Lee H, Pak JN, Shim BS, Kim SH. Reactive oxygen species and p53 mediated activation of p38 and caspases is critically involved in kaempferol induced apoptosis in colorectal cancer cells. J Agric Food Chem. 2018;66(38):9960–7. doi: 10.1021/acs.jafc.8b02656 30211553

[44] Basak D, Uddin MN, Hancock J. The role of oxidative stress and its counteractive utility in colorectal cancer (CRC). Cancers (Basel). 2020;12(11):3336. doi: 10.3390/cancers12113336 33187272 PMC7698080

[45] Liu P-F, Hu Y-C, Kang B-H, Tseng Y-K, Wu P-C, Liang C-C, et al. Expression levels of cleaved caspase-3 and caspase-3 in tumorigenesis and prognosis of oral tongue squamous cell carcinoma. PLoS One. 2017;12(7):e0180620. doi: 10.1371/journal.pone.0180620 28700659 PMC5503265

[46] Perillo B, Di Donato M, Pezone A, Di Zazzo E, Giovannelli P, Galasso G, et al. ROS in cancer therapy: the bright side of the moon. Exp Mol Med. 2020;52(2):192–203. doi: 10.1038/s12276-020-0384-2 32060354 PMC7062874

[47] Jakubowska K, Guzińska-Ustymowicz K, Famulski W, Cepowicz D, Jagodzińska D, Pryczynicz A. Reduced expression of caspase-8 and cleaved caspase-3 in pancreatic ductal adenocarcinoma cells. Oncol Lett. 2016;11(3):1879–84. doi: 10.3892/ol.2016.4125 26998093 PMC4774510

[48] Noble P, Vyas M, Al-Attar A, Durrant S, Scholefield J, Durrant L. High levels of cleaved caspase-3 in colorectal tumour stroma predict good survival. Br J Cancer. 2013;108(10):2097–105. doi: 10.1038/bjc.2013.166 23591201 PMC3670501

