# Supplementary material for: CRISPR/Cas9 mediated ENT2 gene knockout altered purine catabolic pathway and induced apoptosis in colorectal cell lines
Source: PLoS One. 2025 Aug 18;20(8):e0329501. doi: 10.1371/journal.pone.0329501 (PMC12360568; doi:10.1371/journal.pone.0329501)
Supplement: S1 Table — (PDF) [file pone.0329501.s001.pdf]

S1 Table: List of sequencing primers used in qRT- PCR

Gene Name		Primer sequences (5'-3')	Length (bp)	Product length	NCBI Ref. No.
GAPDH	F	GCATCCTGGGCTACACTGAG	20	233	NM_001357943.2
	R	TCCTCTTGTGCTCTTGCTGG	20		
HPRT1	F	GAGTCCTATTGACATCGCCAGT	22	187	NM_000194.3
	R	TCCGCCCAAAGGGAACTGAT	20		
ENT2	F	CCACTCTCTCACCGAAGCCTAA	22	169	NM_001300868.1
	R	GCAGGAAGAACAGCACCAACA	21		
Bcl-2	F	CCAAGGGGGAAACACCAGAA	20	220	NM_000657.3
	R	TCTCCCGGTTATCGTACCCT	20		
BAK	F	TCTCTGGGACCTCCTTAGCC	20	143	NM_001188.4
	R	GTGGGAATGGGCTCTCACAA	20		
Caspase-3	F	AGAACTGGACTGTGGCATTGA	21	174	NM_004346.4
	R	TTCAGCATGGCACAAAGCGA	20		
Caspase-9	F	TGGTGATGTCGGTGCTCTTG	20	152	NM_001229.5
	R	AGTCGATGTTGGAGCCAGTG	20		
P 53	F	GAACAGCTTTGAGGTGCGTG	20	160	NM_001276761.3
	R	CTTCTTTGGCTGGGGAGAGG	20		
