# Supplementary material for: CRISPR/Cas9 mediated ENT2 gene knockout altered purine catabolic pathway and induced apoptosis in colorectal cell lines
Source: PLoS One. 2025 Aug 18;20(8):e0329501. doi: 10.1371/journal.pone.0329501 (PMC12360568; doi:10.1371/journal.pone.0329501)
Supplement: S2 Table — (ZIP) [file pone.0329501.s002.zip › S2A_table.pdf]

S2A Table: sgRNAs sequence targeting the ENT 2 gene (sgENT2s).

Constructs	sgRNA Sequence (Top)	Complementary sgRNA sequence (Bottom)	PAM Sequence	Exon no.
sgNTC	CACCGGAGTGTCGTCGTTGCTCCTAGT	TAAAACTAGGAGCAACGACGACACTCC	-	-
sgENT2-1	CACCGATCCTGAGCACCAACCACACGT	TAAAACGTGTGGTTGGTGCTCAGGATC	GGG	3
sgENT2-2	CACCGCAGGCTCAGGTAACACACGAGT	TAAAACTCGTGTGTTACCTGAGCCTGC	TGG	6
sgENT2-3	CACCGGAAGAGGGTGCTGTAGGTGGGT	TAAAACCCACCTACAGCACCCTCTTCC	AGG	5
sgENT2-4	CACCGCATGGACAGGAGCATGGCAAGT	TAAAACTTGCCATGCTCCTGTCCATGC	GGG	5
sgENT2-5	CACCGTCAACTCCTTCCTGTACCAGGT	TAAAACCTGGTACAGGAAGGAGTTGAC	TGG	3
