# Supplementary material for: CRISPR/Cas9 mediated ENT2 gene knockout altered purine catabolic pathway and induced apoptosis in colorectal cell lines
Source: PLoS One. 2025 Aug 18;20(8):e0329501. doi: 10.1371/journal.pone.0329501 (PMC12360568; doi:10.1371/journal.pone.0329501)

S2B Schematic: Schematic Showing sgENT2s Designs and Their Target Sequence Location Relative to the Entire ENT2 Gene Structure

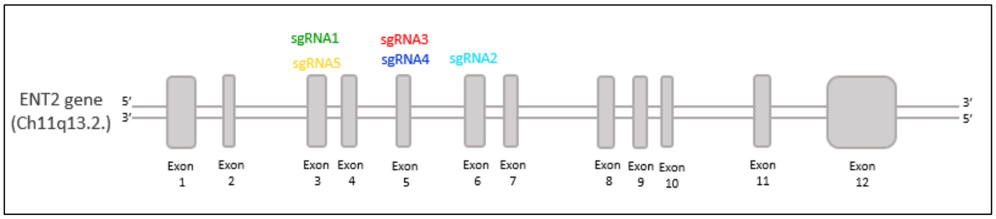

Supplement: S2 Table — (ZIP) [file pone.0329501.s002.zip › S2B_schematic.pdf]
