# Supplementary material for: CRISPR/Cas9 mediated ENT2 gene knockout altered purine catabolic pathway and induced apoptosis in colorectal cell lines
Source: PLoS One. 2025 Aug 18;20(8):e0329501. doi: 10.1371/journal.pone.0329501 (PMC12360568; doi:10.1371/journal.pone.0329501)

S3 Fig: Sanger Sequencing Verification for the Ligated sgENT2 Constructs. 1: sgENT2-1; 2: sgENT2-2: 3: sgENT2-3; 4: sgENT2-4; 5: sgENT2-5.

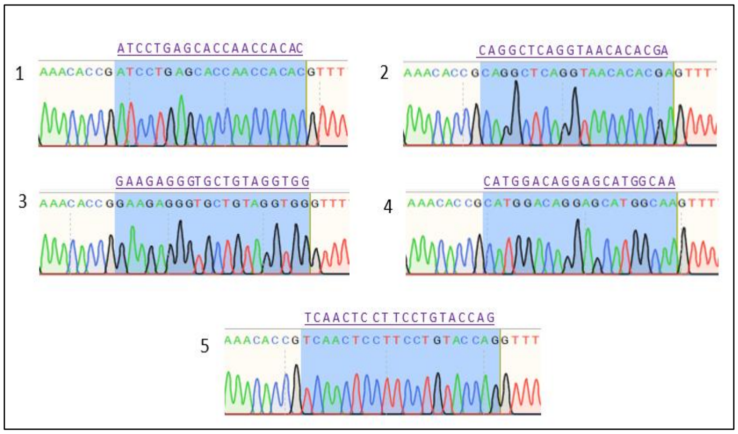

Supplement: S3 Fig — (PDF) [file pone.0329501.s003.pdf]
