# Supplementary figures and images for: CRISPR/Cas9 mediated ENT2 gene knockout altered purine catabolic pathway and induced apoptosis in colorectal cell lines
Source: PLoS One. 2025 Aug 18;20(8):e0329501. doi: 10.1371/journal.pone.0329501 (PMC12360568; doi:10.1371/journal.pone.0329501)

**S4 Fig:** Western Blot full-length image of the positive edit clones of both HT29/KO and DLD1/KO.

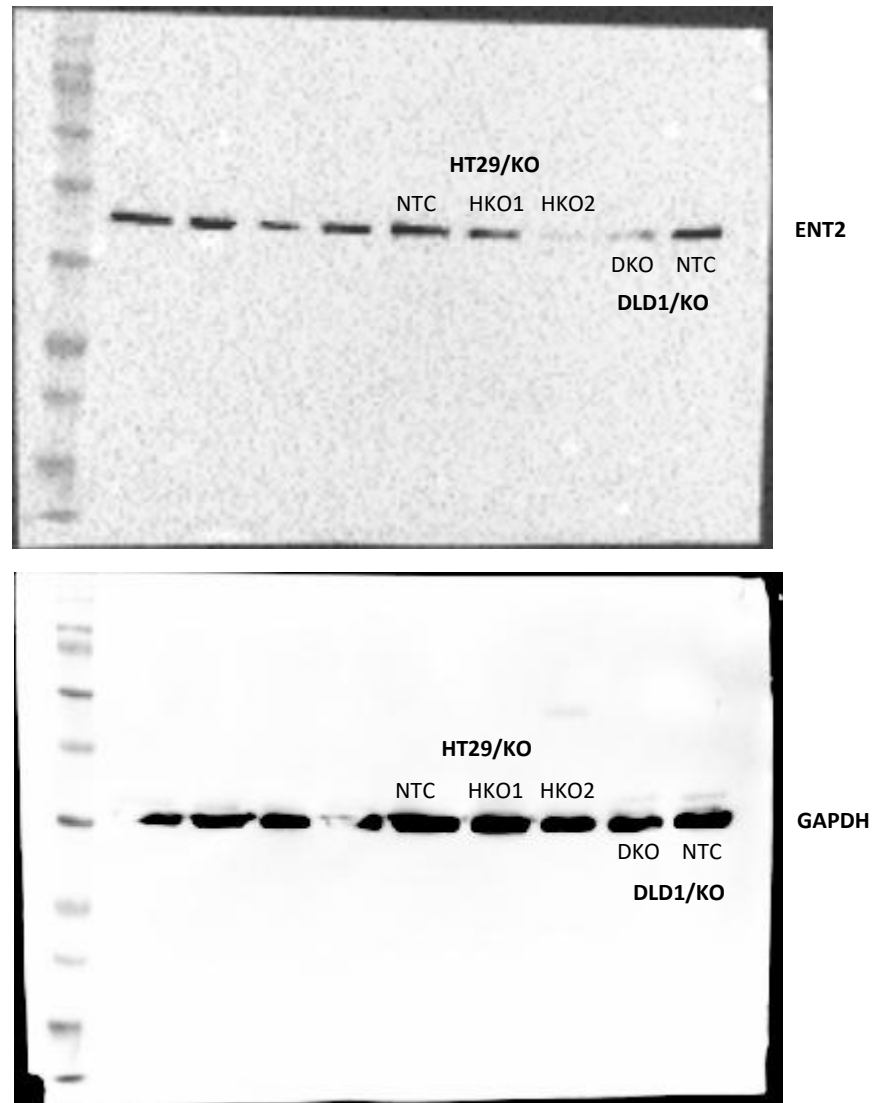

Supplement: S4 Fig — (PDF) [file pone.0329501.s004.pdf]
