# Supplementary material for: CRISPR/Cas9 mediated ENT2 gene knockout altered purine catabolic pathway and induced apoptosis in colorectal cell lines
Source: PLoS One. 2025 Aug 18;20(8):e0329501. doi: 10.1371/journal.pone.0329501 (PMC12360568; doi:10.1371/journal.pone.0329501)

S5 Fig: PCR forward and reverse primers flanking the sgENT2-4 genomic target site. The primers binding sites at the ENT2 genomic region were visualized using the SnapGene viewer program.

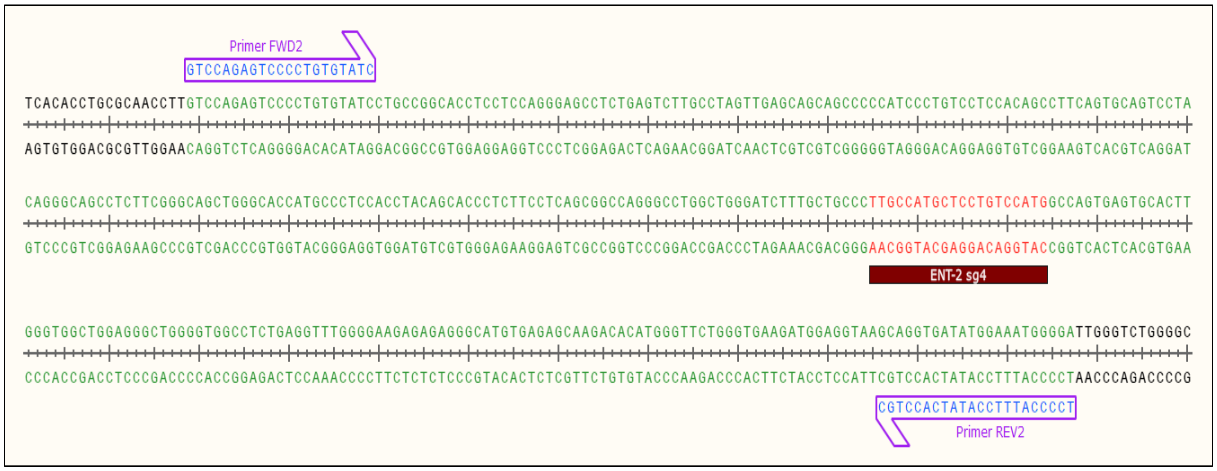

Supplement: S5 Fig — (PDF) [file pone.0329501.s005.pdf]
